# Differences in Patient Characteristics and Midterm Outcome Between Asian and European Patients Treated with Radiofrequency Ablation for Hepatocellular Carcinoma

**DOI:** 10.1007/s00270-016-1462-7

**Published:** 2016-09-26

**Authors:** Mark Christiaan Burgmans, Chow Wei Too, Marta Fiocco, Annarein J. C. Kerbert, Richard Hoau Gong Lo, Jelte J. Schaapman, Arian R. van Erkel, Minneke J. Coenraad, Bien Soo Tan

**Affiliations:** 1 Department of Radiology, Leiden University Medical Centre, Postal Zone C2-S, Albinusdreef 2, 2300 RC Leiden, The Netherlands; 2 Department of Diagnostic Radiology, Singapore General Hospital, Outram Road, Singapore, 169608 Singapore; 3 Department of Medical Statistics and Bioinformatics, Leiden University Medical Centre, Albinusdreef 2, 2300 RC Leiden, The Netherlands; 4 Institute of Mathematics, Leiden University Medical Centre, Leiden University, Niels Bohrweg 1, 2333 CA Leiden, The Netherlands; 5 Department of Hepatology and Gastroenterology, Leiden University Medical Centre, Albinusdreef 2, 2300 RC Leiden, The Netherlands

**Keywords:** Hepatocellular carcinoma, Radiofrequency ablation, Asian, European, Recurrence, Survival

## Abstract

**Purpose:**

The aim of this study was to compare patient characteristics and midterm outcomes after RFA for unresectable Hepatocellular carcinoma (HCC) in Asian and European cohorts.

**Materials and Methods:**

The study was based on retrospective analysis of 279 patients (mean 64.8 ± 12.1 years; 208 males) treated with RFA for de novo HCC in tertiary referral centers in Singapore and the Netherlands, with median follow-up of 28.2 months (quartiles: 13.1–40.5 months). Cumulative incidence of recurrence and death were analyzed using a competing risk model.

**Results:**

Age was higher in the Asian group: 66.5 versus 60.1 years (*p* < 0.0001). The most common etiology was hepatitis B in the Asian group (48.0 %) and alcohol-induced cirrhosis in Europeans (54.4 %); *p* < 0.001. Asian patients had less advanced disease: 35.5, 55.0, and 3.0 %, respectively, had BCLC 0, A, and B versus 21.5, 58.2, and 15.2 % in the European group (*p* = 0.01). The cumulative incidences of recurrence in the Asian group at 1, 2, 3, and 5 years were 37.0, 56.4, 62.3, and 67.7 %, respectively, compared to 32.6, 47.2, 49.7, and 53.4 % in the European group (*p* = 0.474). At 1, 2, 3, and 5 years, the cumulative incidence rates of death in the Asian group were 2.0, 3.9, 4.9, and 4.9 %, respectively, corresponding to 7.7, 9.2, 14.1, and 21.0 % in the European group (*p* = 0.155).

**Conclusion:**

Similar short-term treatment outcomes are achieved with RFA in HCC patients in the South-East Asian and Northern-European populations. Midterm recurrence and death rates differ between the groups as a result of differences in baseline patient characteristics and patient selection. Our study provides insight relevant to the design of future international studies.

## Introduction

Hepatocellular carcinoma (HCC) is a heterogenous condition with multiple variables affecting the course of the disease. The prognosis is not only determined by the tumor burden, but also by the liver function and performance status of a patient. In order to have stratification and prognostication ability, most staging systems have incorporated various prognostic factors [1–6]. The Barcelona Clinic Liver Cancer (BCLC) classification system is the most widely adopted staging system for HCC worldwide and is endorsed by the European Association for the Study of the Liver (EASL) and American Association for the Study of Liver Disease [7, 8]. The Asian Pacific Association for the Study of the Liver (APASL) guidelines are based on results from many of the randomized controlled trials and cohort studies that were also used to devise the BCLC schedule, and both guidelines use similar eligibility criteria for RFA [9]. Despite adherence to similar treatment guidelines, outcomes in daily clinical practice are unlikely to be the same in different parts of the world as a result of geographic differences in characteristics and etiology of HCC. In East-Asia, the incidence rates of HCC are high, and most HCC cases are attributable to chronic hepatitis B infection [7, 10]. In Northern-European countries, HCC is not prevalent, and chronic hepatitis C and alcohol-induced liver disease are the most dominant predisposing risk factors [7, 10].

Prospective clinical trials have been essential in the development of treatment guidelines, but often only recruit patients from a particular region and according to strict eligibility criteria. Real-world observational studies are needed to provide insight into how the implementation of HCC guidelines has affected patient care in different geographic regions. The aim of our descriptive study was to compare the patient characteristics and midterm outcomes after RFA for unresectable, de novo HCC in Asian and European patient cohorts. In this retrospective study, the cumulative incidence rates of recurrence and death after RFA were analyzed in large centers both in South-East Asia and Northern Europe.

## Methods

### Patients

We conducted a retrospective analysis of a patient cohort in a high-volume hospital in Singapore and the Netherlands. Both institutions were tertiary referral centers with dedicated care for hepato-biliary diseases and liver transplant programs. The local medical ethics committee of both institutions approved the retrospective study, and informed consent was waived for the analysis. Between January 2009 and March 2014, 442 consecutive patients were treated with percutaneous RFA for unresectable HCC in the radiology department of one of the two centers. Of the 442 patients, 163 had undergone previous HCC treatment, i.e., ablation, resection, transplantation or transarterial chemoembolization, and these were excluded from the analysis. All remaining 279 patients [mean age ± standard deviation (SD): 64.8 ± 12.1 years; 208 males] were treated with RFA because of newly diagnosed HCC. The diagnosis was based on either tumor histology (*n* = 30) or on radiological imaging criteria according to guidelines by the EASL or the APASL (*n* = 249) [7, 9]. For radiological confirmation of the diagnosis, multiphase contrast-enhanced computed tomography (CECT) and/or dynamic gadolinium-enhanced magnetic resonance imaging (GE-MRI) was used. Arterial hyperenhancement of a lesion with wash-out in the delayed phase was considered to be diagnostic of HCC in patients with liver cirrhosis or chronic hepatitis B/C.

Similar eligibility criteria were used in both centers for local ablation, and these were in accordance with the BCLC and APASL treatment guidelines: a single tumor measuring ≤5 cm or a maximum of 3 HCCs measuring ≤3 cm each and Child Pugh A or B status (7,9). In exceptional cases, RFA was offered also outside BCLC and APASL criteria. In patients with two tumors, RFA was considered if only one HCC measured more than 3 cm and no more than 5 cm. Patients with Child Pugh C who were on the waiting list for liver transplantation could undergo RFA as a bridging therapy to transplantation. Contra-indications for RFA were significant and uncorrectable coagulopathy, extrahepatic metastasis, or macrovascular invasion, and severe liver dysfunction (Child Pugh C) in a patient *not* eligible for liver transplantation.

### Radiofrequency Ablation

All patients gave informed consent prior to treatment. Percutaneous RFA was performed using ultrasound and/or CT guidance. In the European center, procedures were performed under general anesthesia. Local anesthesia and conscious sedation with midazolam and fentanyl were used in the Asian center.

Both centers used similar RFA equipment: either a single electrode was used (3-cm-exposed tip Cooltip (Covidien, Gosport Hamspire, UK) or multiple electrodes with a switch-control system (3- or 4-cm-exposed tip Cooltip). Ablation was performed for 12 (single Cooltip electrode) or 16–20 min (multiple Cooltip electrodes) using standard impedance controlled ablation. In the European center, CECT was performed immediately after ablation on a spiral CT (Aquilion 16, Toshiba, Tokyo, Japan). If this CT showed residual tumor enhancement, immediate re-ablation was performed. In the Asian center, CECT was performed 1 day after ablation (Aquillion 64, Toshiba, Tokyo, Japan). If the CECT showed residual tumor enhancement, re-ablation was performed during the same or subsequent admission, dependent on the patient’s preference.

### Follow-Up

All patients were scheduled for follow-up examinations every 3 months after RFA, including liver function tests and multiphase CECT or dynamic GE-MRI. In the European center, these examinations were also performed at 6 weeks after RFA.

Recurrence was defined as local tumor progression (LTP) and/or a new intrahepatic tumor distant from the treated tumor. Recurrence was distinguished from incomplete ablation. Tumor enhancement on the CECT performed immediately or 1 day after RFA, was classified as incomplete ablation and treated with repeated RFA until complete radiological ablation was achieved. Patients were followed until last follow-up date, death, or till the end of the study.

The median follow-up for all patients was 28.2 months (quartiles: 13.1–40.5 months).

### Statistical Analysis

Comparisons between the two groups were done by student t-test for continuous variables and Pearson Chi-Square test for categorical variables using two-sided tests. A competing risk model with recurrence and death as competing events was used to estimate the cumulative incidence of recurrence and death per center. To study the impact of prognostic factors on recurrence, the cause-specific hazard ratios were estimated by employing a Cox proportional hazard regression model with transplantation as time-dependent risk factor [11]. A Cox’s proportional hazard model was employed to study the association between risk factors and overall survival with recurrence and transplantation as time-dependent risk factors. A difference was considered significant when *p* < 0.05. The statistical analyses were performed using SPSS 21 (IBM, Armonk, NY, USA). The competing risks analysis was performed in the R-software environment with the mstate library [12, 13].

## Results

### Patient Characteristics

Baseline demographics of all patients are shown in Table 1. The median age of patients in the Southeast Asian group was slightly higher than that of the Northern European patients (*p* < 0.0001). Statistically significant differences between the patient groups were also seen in underlying liver disease and BCLC stage (*p* < 0.0001 and *p* = 0.01, respectively). In the European patients, alcoholic liver disease was most prevalent (54.4 %) followed by hepatitis C (22.7 %), whereas the majority of Asian patients suffered from chronic hepatitis B (48.0 %). The percentage of patients without underlying liver disease was much higher in the Asian group compared with the European group: 19.0 versus 6.3 %. The Asian group had a higher percentage of patients with BCLC very early stage: 35.5 versus 21.5 % in the European group. Both the percentages of patients with BCLC early stage and intermediate stage were higher in the European group: 58.2 and 15.2 %, respectively, versus 55.0 and 3.0 % in the Asian group. These differences in BCLC stage may be explained by the dissimilarities in Child Pugh class, number of tumors, and maximal tumor diameter between the two groups. In the Asian group, a higher percentage of patients had Child Pugh A status (68.5 vs. 60.8 %), a single tumor (77.0 vs. 67.1 %), and the mean maximal diameter of the largest tumor was smaller (23.7 ± 11.3 vs. 26.8 ± 12.6 mm). The differences in Child Pugh status, tumor number and tumor size did not reach statistical significance.

**Table 1:** Baseline characteristics of 279 patients treated with RFA for de novo HCC

	Asia–Pacific; *n* = 200 (%)	European; *n* = 79 (%)	Total; *n* = 279 (%)	*p* value
Age (years), mean ± SD	66.5 ± 10.7	60.1 ± 14.3	64.8 ± 12.1	**<0.0001**
Male/female	144/56 (72.0/28.0)	64/15 (81.0/19.0)	208/71 (74.6/25.4)	0.78
Etiology				**<0.0001**
HBV	96 (48.0)	7 (8.9)	103 (36.9)	
HCV	26 (13.0)	18 (22.7)	44(15.8)	
Alcohol	21 (10.5)	43 (54.4)	64 (22.9)	
NASH	11 (5.5)	3 (3.8)	14 (5.0)	
Cryptogenic	38 (19.0)	5 (6.3)	43 (15.4)	
Others		3 (3.8)	11 (3.9)	
AFP (ng/mL), mean ± SD	141.4 ± 753.3^a^	346.9 ± 1600.6^b^	212.7 ± 1122.7	0.289
Child pugh class				0.248
A	137 (68.5)	48 (60.8)	185 (66.3)	
B	49 (24.5)	27 (34.2)	77 (27.6)	
C	14 (7.0)	4 (5.0)	17 (6.1)	
Number of tumors				0.139
1	154 (77.0)	53 (67.1)	207 (74.2)	
2	39 (19.5)	24 (30.4)	63 (22.6)	
3	7 (3.5)	2 (2.5)	9 (3.2)	
Maximal diameter largest tumor (mm), mean ± SD	23.7 ± 11.3	26.8 ± 12.6	24.9 ± 12.5	0.85
Maximal diameter largest tumor				0.106
<10 mm	9 (4.5)	0 (0.0)	9 (3.2)	
10 to <20 mm	84 (42.0)	26 (31.9)	110 (39.4)	
20 to 30 mm	61 (30.5)	26 (32.9)	87 (31.2)	
>30 mm	46 (23.0)	27 (34.2)	73 (26.1)	
BCLC stage				**0.01**
0	71 (35.5)	17 (21.5)	88 (31.5)	
A	110 (55.0)	46 (58.2)	156 (55.9)	
B	6 (3.0)	12 (15.2)	18 (6.5)	
C	0 (0.0)	0 (0.0)	0 (0.0)	
D	13 (6.5)	4 (5.0)	17 (6.1)	

Statistically significant *p* values are given in bold (*p* < 0.05)

*HBV* hepatitis B virus, *HCV* hepatitis C virus, *NASH* nonalcoholic steatosis hepatitis, *BCLC* Barcelona Clinic Liver Cancer

^a^55 missing

^b^2 missing

### Treatment Outcome

In 269 patients (96.4 %), technical success was achieved after a single RFA procedure. In the remaining 10 patients, a second ablation procedure was needed to achieve technical success.

**Fig. 1:**
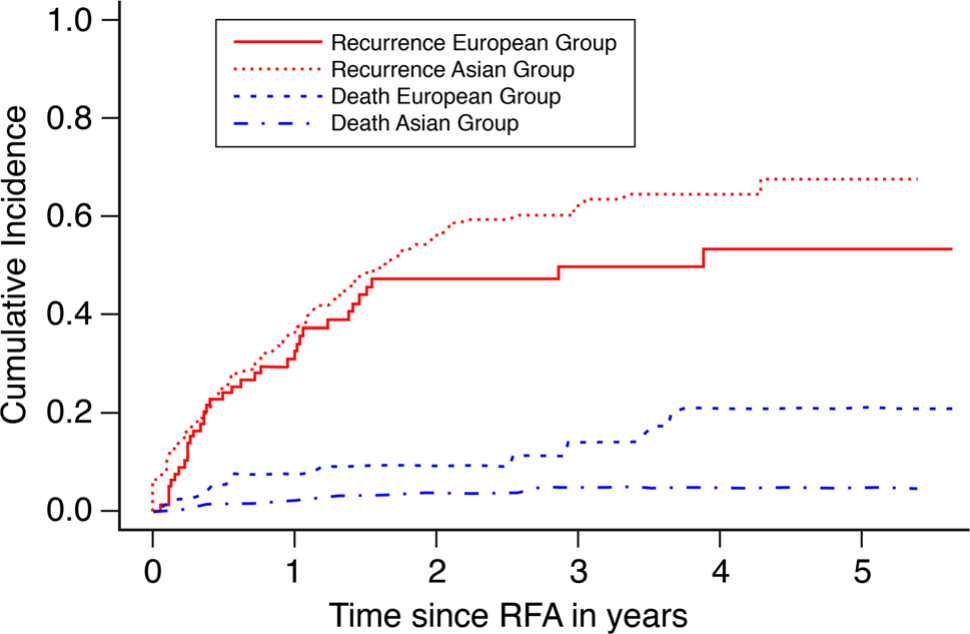
Cumulative incidence of recurrence and death in the South-East Asian and Northern European patient group

The cumulative incidence of recurrence showed a similar trend in both the Asian and European groups during the first 1.5 years after RFA (see Fig. 1). At 6, 12, and 18 months, the cumulative incidence rates for recurrence in the Asian group were equal to 25.5 % (95 % CI 19.5–31.6), 37.0 % (95 % CI 30.3–43.8), and 49.1 % (95 % CI 41.9–56.2), respectively, compared to 24.1 % (95 % CI 14.7–33.5), 32.6 % (95 % CI 22.0–43.2), and 45.5 % (95 % CI 33.8–57.2), respectively, in the European group. The cumulative incidence of recurrence was higher in the Asian group at 2, 3, and 5 years: 56.4 % (95 % CI 49.1–63.8), 62.3 % (95 % CI 54.7–69.8), and 67.7 % (95 % CI 58.6–76.7), respectively, compared to 47.2 % (95 % CI 35.4–59.0), 49.7 % (95 % CI 37.5– 62.0), and 53.4 % (95 % CI 40.2–66.6), respectively, in the European group. The difference between the cumulative incidences of recurrence for the two groups was not significant (*p* = 0.474).

The cumulative incidence of death was higher in the European population compared with the Asian group (Fig. 1). At 1, 2, 3, and 5 years, the cumulative incidence rates of death were 2.0 % (95 % CI 0.06–4.0), 3.9 % (1.0–6.7), 4.9 % (1.5–8.3), and 4.9 % (1.5–8.3), respectively, in the Asian group and 7.7 % (1.8–13.6), 9.2 % (2.7–15.8), 14.1 % (5.1–23.1), and 21.0 % (9.0–33.1) in the European group. The differences in cumulative death between the two groups did not reach statistical significance (*p* = 0.155).

### Prognostic Factors Associated with the Risk of Recurrence

A maximal tumor diameter >3 cm and tumor number >1 were independent risk factors for recurrence after RFA (see Table 2). The cause-specific hazard ratio (csHR) was equal to 1.568 (95 % CI 1.083–2.271) for patients with HCCs >3 cm. Patients with more than 1 tumor were 1.5 times more likely to develop recurrence than patients with a single tumor (HR_c_ 1.494 (95 % CI 1.031–2.163). Liver transplantation had a significant protective effect on tumor recurrence (HR_c_ 0.065; 95 % CI 0.009–0.480).

**Table 2:** Cause-specific hazard ratios to evaluate the effect of prognostic factors on risk of recurrence (multivariate analysis)

	csHR	95.0 % CI for HR		*p* value
		Lower	Upper	
Female (reference male)	.844	.567	1.256	0.403
Child Pugh A (reference)				0.480
Child Pugh B	.795	.535	1.181	0.256
Child Pugh C	.810	.412	1.591	0.540
Largest tumor diameter >3 cm	1.568	1.083	2.271	**0.017**
Tumor number >1	1.494	1.031	2.163	**0.034**
Hepatitis B (reference)				0.739
Hepatitis C	.857	.497	1.477	0.578
Alcohol-induced	1.175	.702	1.967	0.538
Other	1.100	.692	1.750	0.686
South-East Asian center^a^	.978	.609	1.568	0.925
Liver transplantation	.065	.009	.480	**0.007**

Statistically significant *p* values are given in bold (*p* < 0.05)

*csHR* cause-specific hazard ratio, *CI* confidence interval

^a^The Northern-European Center was used as the reference center

### Cox Regression Model for Overall Survival

Child Pugh B/C status and recurrence were independent risk factors for death after RFA (see Table 3). The hazard ratios (HRs) for Child Pugh B and C were equal to 2.924 (95 % CI 1.582–5.404) and 4.824 (95 % CI 2.100–11.083), respectively, with Child Pugh A status as reference category. The HR was almost 5 times increased in patients with recurrence compared with patients without recurrence (HR 4.524; 95 % CI 2.438–8.395). An increased HR of death was found in patients with either hepatitis C or alcohol-induced liver disease compared to those with hepatitis B, but the differences were nonsignificant.

**Table 3:** Hazard ratios to evaluate the effect of prognostic factors on overall survival (multivariate analysis)

	HR	95.0 % CI for HR		*p* value
		Lower	Upper	
Female (reference male)	.968	.491	1.911	0.926
Child Pugh A (reference)				
Child Pugh B	2.924	1.582	5.404	**0.001**
Child Pugh C	4.824	2.100	11.083	**0.000**
Largest tumor diameter >3 cm	1.326	.714	2.462	0.372
Tumor number >1	.679	.355	1.299	0.242
Hepatitis B (reference)				0.399
Hepatitis C	1.573	.697	3.549	0.275
Alcohol-induced	1.234	.554	2.747	0.607
Other	.776	.326	1.845	0.566
South-East Asian center	.531	.269	1.049	0.068
Recurrence (time-dependent)	4.524	2.438	8.395	**0.000**
Liver transplantation	.805	.318	2.036	0.647

Statistically significant *p* values are given in bold (*p* < 0.05)

*HR* hazard ratio, *CI* confidence interval

Liver transplantation had a protective effect, though not statistically significant (HR 0.805; 95 % CI 0.318–2.036).

### Further Treatment

Table 4 provides an overview of consecutive treatments that were administered in patients with recurrent disease. No significant differences were seen between the two groups other than the higher proportion of patients in the European group receiving a liver transplantation. In the European group, 44.3 % (*n* = 35) of patients eventually underwent liver transplantation compared to 3.0 % in the Asian group (*n* = 6) (*p* < 0.0001).

**Table 4:** Summary of second-line treatment in the South-East Asian and Northern-European patient cohorts

Second-line treatment	Asian group; *n* = 97 (48.5 %)	European group; *n* = 54 (68.4 %)	*p* value
Resection	8 (4.0)	2 (2.5)	0.552
RFA	68 (34.0)	21 (26.6)	0.231
TACE/TARE	35 (17.5)	13 (16.5)	0.835
Liver transplantation	5 (2.5)	35 (44.3)	**<0.0001**
Sorafenib	3 (1.5)	4 (5.1)	0.89

Statistically significant *p* value is given in bold (*p* < 0.05)

*TACE* transarterial chemoembolization, *TARE* transarterial radioembolization

## Discussion

Our study provides insight into the differences in baseline characteristics and treatment outcomes between a South-East Asian and a Northern-European cohort of patients undergoing RFA for de novo HCC. The differences observed may have implications for clinical management and the design of large multicenter, international studies.

Our study confirms that hepatitis B is the leading cause of HCC in South-East Asia, whereas most HCC cases in the Northern Europe are related to alcohol or hepatitis C. This is well known from the literature [7, 10]. The higher percentage of patients without known risk factors in the Asian study group is also consistent with previous reports [14].

In the Asian group, the number of tumors as well as Child Pugh score was lower compared with the European group. These observed dissimilarities between the two groups may, in part, reflect differences in patient selection. First of all, differences in screening between the Netherlands and Singapore may have resulted in detection of tumors at an early stage in the Asian group. In both countries, six-monthly screening with ultrasonography was common practice during the study period, but the higher incidence of HCC in Asia is likely to result in higher awareness and better adherence to the screening program by Singaporean doctors and patients. Second, differences in baseline characteristics may be a result of differences in the EASL and APASL guidelines. According to the APASL guidelines, the diagnosis of HCC can be made regardless of the size of a lesion, if a lesion has typical arterial enhancement and portovenous ‘wash-out’ on diagnostic imaging. This is different from the EASL guidelines that state that noninvasive criteria only apply in patients with typical lesions >1 cm. The difference in diagnostic criteria between the APASL and EASL guidelines explains the difference in baseline tumor size between the Asian and European groups in our study. In the Asian group, 9 patients had a maximal tumor diameter of <1 cm, whereas all European patients had a tumor larger than 1 cm. This is also reflected by the smaller mean tumor diameter of patients in the Asian group compared to that of the European patients (23.7 ± 11.3 vs. 26.8 ± 12.6 respectively). As the noninvasive diagnostic accuracy is lower in lesions <1 cm, there is an increased risk of a false-positive diagnosis of HCC in the Asian group in our study. It is unlikely, however, that this had a significant impact on the results of our study, as only 4.5 % of patients in the Asian group had lesions <1 cm.

There is considerable overlap between the BCLC and APASL treatment algorithms with regard to selection of patients for RFA. According to both algorithms, eligible candidates are Child Pugh A/B patients with a single tumor ≤5 cm or up to three nodules of ≤3 cm each *and* the absence of vascular invasion of extrahepatic disease (the EASL guidelines do not clearly give a maximal diameter for a solitary tumor, but 5 cm is generally considered the limit beyond which RFA is associated with unacceptable high recurrence rates). The EASL and APASL guidelines both recommend RFA as an alternative to resection for patients not suitable for surgery, but do not use the same criteria to select surgical candidates [15]. The EASL guidelines recommend resection for patients with a single tumor with very well-preserved liver function, defined as normal bilirubin with either hepatic vein pressure gradient <10 mmHg or platelet count ≥100 × 10^9^/L. According to the APASL guidelines, surgical resection should be considered for single or multifocal disease, anatomically resectable, and with satisfactory liver function reserve without strict cutoff values. As a result of the more conservative criteria for resection in the EASL guidelines, patients may have been referred for ablation in the European center, whereas the same patients may still have been surgical candidates in the Asian institution. This may have contributed to a higher percentage of patients in the European group with Child Pugh B status and >1 tumor. Following the APASL guidelines, decisions on resectability in South-East Asia are more contingent on age and functional capacity of a patient. This may also explain the significantly higher age of patients in the Asian cohort.

The differences in cumulative incidence of recurrence and death between the Asian and European groups are likely related to a multitude of variables, such as patient selection, baseline patient characteristics, pathogenesis and histopathology of tumors, differences in clinical management, and treatment of underlying liver disease. Patients in the European group had an insignificant higher midterm cumulative incidence of death. As the recurrence rate in the European patients was not higher than that in the Asian patients, the poorer survival rate is probably attributable to factors other than disease progression. It is likely that the significantly higher baseline Child Pugh score had a negative impact on survival. A higher Child Pugh score has been shown to be associated with poorer overall survival in previous studies [16–23]. The lower Child Pugh score may also reflect a difference between the two groups in the proportion of patients with cirrhosis, as the development of HCC in the absence of cirrhosis tends to be more common in Asian patients. Another factor could be the differences in therapeutic options for the underlying liver disease. Antiviral agents such as lamivudine, adefovir dipivoxil, or entecavir may improve overall survival after RFA in hepatitis B patients, whereas therapeutic options for hepatitis C and alcohol-induced liver cirrhosis were limited during the study period [24, 25]. Finally, differences in molecular pathogenesis of HCC between regions and races may result in differences in outcome [26].

Although previous studies have shown that liver transplantation improves survival in patients with HCC, such a survival benefit was not found in our study [27]. Transplantation did have a significant protective effect on tumor recurrence, but the protective effect on survival did not reach statistical significance. This is likely to be related to the relatively small number of patients that were transplanted in our study (14.3 %).

Our study findings are of importance when interpreting published studies on RFA in HCC patients. Comparison of studies that have been conducted in different parts of the world is complicated by the differences in patient characteristics, selection, and clinical management. Results obtained in an Asian population cannot be extrapolated to a European population without notion of these differences, and vice versa. Our results may also have important implications for the design of new international studies. Based on our results, the impact of RFA on survival may be more difficult to determine in a Northern-European population than in a South-East Asian cohort as factors other than tumor progression play a more important role in the first group of patients. European patients eligible for RFA are likely to have risk factors other than tumor recurrence that are associated with poorer survival, such as hepatitis C or alcohol-induced liver cirrhosis, and higher BCLC stage. To demonstrate survival benefit of RFA in a group of European patients with unresectable HCC, one may thus need a larger sample size than that in an Asian patient group.

Our descriptive study has several limitations. The first limitation is the retrospective design of the study. Second, the numbers of centers included in our analysis are limited, and therefore the data may not be representative for all centers in the geographic regions that were compared. Third, some predicting factors that may have have been different between the two cohorts were not analyzed, for example, co-morbidity, tumor histology, and antiviral treatment of hepatitis. Fourth, a small number of patients in the Asian and European groups were treated outside APASL and EASL criteria, respectively. This may have caused differences between the groups that are not attributable to differences in the regional guidelines. Finally, we did not analyze the cause of death. The poorer survival rate in the European patients may have been related to causes other than progression of tumor or underlying liver disease. It is not unlikely that the proportions of patients with tobacco abuse and poor nutritional status were higher in the European group given the higher prevalence of alcohol abuse.

In conclusion, the baseline characteristics of patients treated with RFA for de novo HCC differ between Northern-European and South-East Asian patients. Despite these differences, similar short term treatment outcomes are achieved by applying regional recommendations for RFA in HCC patients. Midterm recurrence and death rates differ between the two groups, and this may be explained by differences in underlying liver disease, screening, and the more conservative approach to resection in European countries.
